# Declining rates of global routine vaccination coverage amidst the COVID-19 syndemic: a serious public health concern

**DOI:** 10.31744/einstein_journal/2021ED6552

**Published:** 2021-12-14

**Affiliations:** 1 Jinnah Medical & Dental College Karachi Sindh Pakistan Jinnah Medical & Dental College, Karachi, Sindh, Pakistan.; 2 University of Sheffield Sheffield United Kingdom University of Sheffield, Sheffield, United Kingdom.; 3 IRCCS Fondazione Don Carlo Gnocchi Milan Italy IRCCS Fondazione Don Carlo Gnocchi, Milan, Italy.; 4 Universidade de São Paulo Faculdade de Medicina de Ribeirão Preto Ribeirão Preto SP Brazil Faculdade de Medicina de Ribeirão Preto, Universidade de São Paulo, Ribeirão Preto, SP, Brazil.

At the end of 2019, a novel coronavirus named severe acute respiratory syndrome coronavirus 2 (SARS-CoV-2), began to spread in the city of Wuhan, China, causing an outbreak of a highly transmissible and potentially severe viral pneumonia. This novel coronavirus disease, known as coronavirus disease 2019 (COVID-19), rapidly advanced all over the globe and was considered a pandemic in March 2020.^( 1 , 2 )^ However, in a recent article, COVID-19 has been redefined as a syndemic, since a background of social and economic disparity increased the adverse effects of the SARS-CoV-2 infection, together with several non-communicable diseases.^( 3 )^ In an effort to reduce the increasing morbidity and mortality rates of COVID-19 across the world, societies are trying to limit the routine utilization of healthcare facilities, to reduce the associated deleterious impacts.^( 4 , 5 )^

The disease has halted many usual activities, such as commerce, travel and healthcare services. Amid global lockdowns, daily healthcare services and elective surgical procedures have been cancelled across multiple healthcare settings. Furthermore, healthcare workers have been retrained and redeployed to prioritize the provision of care to clinically deteriorating COVID-19 patients. Simultaneously, the syndemic has interrupted routine immunization practices across all age groups, particularly for the pediatric population. Delays, reorganization or suspension of these routine vaccinations may lead to a surge in numbers of infections and subsequent deaths due to vaccine-preventable diseases. Part of the population may also become susceptible to diseases that were previously controlled or eradicated.^( 6 )^ Prevention and control measures to avoid spread of COVID-19 have hindered child and mass immunization efforts globally, placing millions of children at risk of other potentially fatal vaccine-preventable diseases.^( 7 )^ The most affected vaccination campaigns include those for measles, poliomyelitis, diphtheria, pertussis, tetanus and meningitis. Therefore, some vaccine-preventable diseases are now making a comeback, thereby putting local populations at risk.^( 8 )^

Some factors, such as parental fear, lockdown measures, prioritizing COVID-19 patients, and logistics delivery issues (such as vaccine transport delays), have greatly contributed to delayed and interrupted immunizations. According to the World Health Organization (WHO), the United Nations International Children's Emergency Fund (Unicef), the Global Vaccine Alliance (GAVI), and the Sabin Vaccine Institute, routine immunization programs have been significantly affected in at least 68 countries, affecting approximately 80 million children. In GAVI-supported low-income countries, an additional 24 million individuals are at risk of missing out on vaccines against measles, poliomyelitis, rotavirus, meningitis, rubella and human papillomavirus.^( 9 )^

In the first five months of the syndemic, numerous countries had suspended their routine immunization campaigns.^( 9 )^ Cessation of vaccination campaigns has led to alarming and potentially devastating public health outcomes, with diphtheria recently reemerging in some countries, such as Venezuela, Pakistan, Nepal, Bangladesh and Yemen, where population displacement greatly effects public health systems. Similarly, resurgence of cholera has been reported in Bangladesh, Cameroon, Mozambique, South Sudan and Yemen.^( 10 )^ Through April 1st to 15, 2020, a complete suspension of routine immunizations was observed in Vietnam, while India's routine immunization program was also disrupted, for health workers were redeployed in an effort to curb the spread of the syndemic.^( 6 )^

As per the Global Polio Eradication Initiative (GPEI), it was recommended suspend the vaccination campaigns against poliomyelitis until the second half of 2020.^( 11 )^ Pakistan also postponed their polio catch-up immunization campaigns until June 1^st^, 2020.^( 6 )^ The syndemic caused the suspension of 46 poliovirus immunization campaigns in 38 countries, most notably in African nations. Recently, over 30 countries reported a mutated vaccine-derived strain of poliovirus,^( 9 )^ and Niger reported a recent polio outbreak.^( 12 )^ Pakistan and Afghanistan have further reported a wild poliovirus type 1, while cases of type 2 poliovirus, mutated from the oral vaccine, have appeared in Chad, Ethiopia, Ghana and Pakistan.^( 13 )^

Furthermore, it is important to consider in the current context that measles, a disease that causes severe morbidity and has a case fatality rate of 0.2%, is far more contagious (basic reproduction rate – R_0_ – of 12-16) than COVID-19.^( 14 )^ Measles weakens the immune system for a long period of time, resulting in immune amnesia, which leaves patients susceptible to other infections.^( 15 )^ In England, during the first three weeks of national lockdown, the numbers of measles, mumps, and rubella vaccines delivered dropped by 20%, with smaller uptake drops were reported amongst children in Scotland.^( 16 )^ Similarly, immunization coverage has simultaneously decreased in the adult population too, due to similar concerns previously described, and the fear of attending healthcare facilities.^( 17 )^ Pneumococcal and influenza vaccines are also being affected in light of COVID-19. A rise of COVID-19 incidence, alongside mortality, has been noticed in patients with chronic lung and cardiovascular diseases as well as diabetes, all of which are recognized risk factors for pneumococcal infections.^( 18 )^ However, there is limited data available regarding influenza's interaction with COVID-19.^( 6 , 19 , 20 )^ In the United States, the coverage of publicly funded vaccines, including hepatitis, meningitis, poliomyelitis and rotavirus, declined drastically in comparison to 2019. In New York City, vaccinations have dropped by an overall average of 63%, rising to 91% for children above 2 years of age, whilst in the state of California, rates have declined by 40%.^( 21 )^ Furthermore, the state of Ohio reported a 45% decline in vaccination rates in its pediatric population. Whilst under normal circumstances, ~1,000 measles shots would be given monthly, in April 2020, only 32 were reported in the state.^( 22 )^

Hesitancy and refusal to accept vaccination have been a serious public health concern for a number of years, both before and during the COVID-19 syndemic. Many barriers to vaccination, most of them centering on concerns about vaccine safety, are well described in the literature.^( 23 )^ However, the reasons for vaccination delay or refusal change over time, as highlighted by this syndemic. Fear mongering should be fought with verified information, as sharing false and non-authenticated news and opinions with vulnerable communities has significantly contributed to skepticism surrounding the development and uptake of vaccines.^( 24 )^ Immunization is amongst the most effective public health measures to prevent the spread of diseases. To raise awareness and fight misinformation, high-level activity is required to preserve ongoing vaccination coverage and limit preventable infections. There is an urgent need to encourage government action, increase support to communities and reassure the public about the importance and safety of attending primary care units for routine vaccination during the COVID-19 syndemic. An effective vaccine is likely to be the most crucial method to control and curb the syndemic, as agreed upon by public health officials globally. A minimum of one to two years is typically needed to develop an effective vaccine,^( 25 – 28 )^ and an efficacious vaccine is deemed vital to prevent morbidity and mortality.^( 29 )^ To date, dozens of candidate SARS-CoV-2 vaccines have or are undergoing clinical trials,^( 30 )^ with a number already approved and being implemented for mass public vaccination.^( 31 , 32 )^ However, Russia and China have been reported to perform SARS-CoV-2 vaccination outside of clinical trials, which has encountered widespread criticism, in light of uncertain safety profiles of these candidate vaccines, without robust safety and efficacy data being made widely available from phase 3 clinical trials.^( 33 , 34 )^

According to the WHO, a “clear demonstration of efficacy (on population basis) ideally with ~50% point estimate”, is the minimum standard for a newly developed vaccine to be accepted for mass use against COVID-19. The efficacy of such a vaccine should be checked against “disease, severe disease, and/or shedding/transmission” endpoints.^( 35 )^ Data for long-term efficacy and safety profiles are also needed. To support the latter, clinical trials of sufficient duration and participant numbers are required to further assess whether the vaccines make COVID-19 more hazardous (the so-called disease enhancement).^( 36 , 37 )^ Evidence is needed to support the use of SARS-CoV-2 vaccines, and prevent the negative impacts on the public acceptance of them, which could hinder the control of COVID-19 spread, as well as deleteriously affect the administration of routine vaccines for other communicable diseases.^( 38 )^

In conclusion, public authorities are setting up fast-track approval processes for effective vaccines against SARS-CoV-2, and strong vaccination campaigns are already underway in several countries. Vaccination hesitancy remains a persistent concern for both the SARS-CoV-2 vaccination and all other routine immunization programs. It is believed that effective public health measures can reduce the impact that vaccine delay or refusal has on routine immunization processes, thereby preventing the reemergence of previously eradicated diseases.
