# Supplementary material for: Declining rates of global routine vaccination coverage amidst the COVID-19 syndemic: a serious public health concern
Source: Einstein (Sao Paulo). 2021 Dec 14;19:eED6552. doi: 10.31744/einstein_journal/2021ED6552 (PMC8687643; doi:10.31744/einstein_journal/2021ED6552)
Supplement: Translation

# Translation: Taxas decrescentes de cobertura global da vacinação de rotina em meio à sindemia da COVID-19: um grave problema de saúde pública

**Affiliations:** 1 Karachi Sindh Pakistan Jinnah Medical & Dental College, Karachi, Sindh, Pakistan.; 2 Sheffield United Kingdom University of Sheffield, Sheffield, United Kingdom.; 3 Milan Italy IRCCS Fondazione Don Carlo Gnocchi, Milan, Italy.; 4 Ribeirão Preto SP Brasil Faculdade de Medicina de Ribeirão Preto, Universidade de São Paulo, Ribeirão Preto, SP, Brasil.

No fim de 2019, um novo coronavírus, denominado coronavírus da síndrome respiratória aguda grave 2 (SARS-CoV-2), começou a se propagar na cidade de Wuhan, na China, causando um surto de pneumonia viral altamente transmissível e potencialmente grave. Essa nova doença, conhecida como doença pelo coronavírus 2019 (COVID-19), avançou rapidamente pelo mundo e passou a ser considerada uma pandemia em março de 2020.^( 1 , 2 )^Contudo, em artigo recente, a COVID-19 foi redefinida como uma sindemia, já que o contexto de desigualdade social e econômica tem aumentado os efeitos adversos da infecção pelo SARS-CoV-2, junto de diversas outras doenças não transmissíveis.^( 3 )^Em uma tentativa de reduzir as taxas crescentes de morbidade e mortalidade da COVID-19 em todo o mundo, as sociedades em geral têm buscado limitar a procura por assistência em estabelecimentos de saúde, objetivando diminuir os efeitos negativos associados.^( 4 , 5 )^

A doença tem levado, por sua vez, à interrupção de vários setores essenciais, como comércio, viagens e serviços de saúde. Diante das medidas de confinamento globais ( *lockdowns* ), os serviços de saúde de rotina, bem como os procedimentos cirúrgicos eletivos, têm sido suspensos em diferentes níveis de atenção à saúde. Além disso, profissionais da área da saúde têm sido capacitados e realocados, priorizando-se o atendimento a pacientes com quadro clínico grave da COVID-19. Ao mesmo tempo, a sindemia tem descontinuado a oferta da vacinação de rotina para todas as faixas etárias, principalmente aquelas voltadas para a população infantil. Os atrasos, a reestruturação ou mesmo a suspensão da vacinação de rotina podem levar a um aumento no número de infecções e mortes causadas por doenças imunopreveníveis. Desse modo, parte considerável da população pode também se tornar suscetível a doenças previamente controladas ou erradicadas.^( 6 )^As medidas de prevenção e controle para evitar a propagação da COVID-19 têm dificultado a continuidade dos programas de vacinação infantil e em massa em todo o mundo, colocando milhões de crianças em risco de contrair outras doenças potencialmente fatais, as quais são preveníveis por imunização.^( 7 )^As campanhas de vacinação mais prejudicadas incluem as de sarampo, poliomielite, difteria, coqueluche, tétano e meningite. Desse modo, algumas doenças imunopreveníveis estão agora ressurgindo, colocando populações locais em risco.^( 8 )^

Fatores como medo parental, medidas de confinamento, priorização de pacientes com COVID-19 e questões logísticas (como atrasos no transporte de vacinas) têm contribuído muito para o atraso e a interrupção das vacinações. De acordo com a Organização Mundial da Saúde (OMS), o Fundo das Nações Unidas para a Infância (Unicef), a *Global Vaccine Alliance* (GAVI) e o *Sabin Vaccine Institute* , os programas de vacinação de rotina têm sido significativamente impactados em pelo menos 68 países, acarretando prejuízos a cerca de 80 milhões de crianças. Em países de baixa renda apoiados pela GAVI, outras 24 milhões de pessoas correm o risco de não se vacinar contra sarampo, poliomielite, rotavírus, meningite, rubéola e papilomavírus humano.^( 9 )^

Nos primeiros 5 meses da sindemia, diversos países suspenderam suas campanhas de vacinação de rotina.^( 9 )^A suspensão das campanhas de vacinação tem levado a resultados de saúde pública alarmantes e potencialmente devastadores, como o ressurgimento da difteria em países como Venezuela, Paquistão, Nepal, Bangladesh e Iêmen, onde o deslocamento de populações exerce um grande impacto sobre os sistemas públicos de saúde. Da mesma forma, a cólera também ressurgiu e, atualmente, há registros em Bangladesh, Camarões, Moçambique, Sudão do Sul e Iêmen.^( 10 )^De 1° a 15 de abril de 2020, houve suspensão total da vacinação de rotina no Vietnã, ao mesmo tempo em que o programa de vacinação de rotina da Índia foi interrompido, dado que profissionais de saúde foram realocados, na tentativa de conter a propagação da sindemia.^( 6 )^

De acordo com a Iniciativa Global de Erradicação da Pólio (GPEI - *Global Polio Eradication Initiative* ), foi estabelecida a recomendação de suspensão das campanhas de vacinação contra a poliomielite até o segundo semestre de 2020.^( 11 )^O Paquistão também adiou suas campanhas de vacinação contra a poliomielite até 1° de junho do mesmo ano.^( 6 )^A sindemia já causou a suspensão de 46 campanhas de vacinação contra o poliovírus em 38 países, sobretudo em nações africanas. Recentemente, mais de 30 países registraram uma cepa de poliovírus mutado, derivado da vacina,^( 9 )^enquanto o Níger registrou um surto recente de poliomielite.^( 12 )^Ambos, Paquistão e Afeganistão registraram ainda circulação do poliovírus selvagem tipo 1 e, paralelamente a isso, casos de poliovírus tipo 2, mutado a partir da vacina oral, ocorreram no Chade, Etiópia, Gana e Paquistão.^( 13 )^

Além disso, é importante ressaltar, no contexto atual, que o sarampo, doença que causa morbidade grave, com taxa de letalidade de 0,2%, é muito mais contagioso (número básico de reprodução – R_0_– de 12-16) que a COVID-19.^( 14 )^O sarampo enfraquece o sistema imunológico por um longo período, resultando em amnésia imunológica e suscetibilidade dos pacientes a outras infecções.^( 15 )^Na Inglaterra, durante as primeiras 3 semanas de quarentena nacional, os números de doses aplicadas de vacinas contra sarampo, caxumba e rubéola diminuíram 20%, e reduções menores foram também registradas entre crianças na Escócia.^( 16 )^Similarmente, a cobertura vacinal também diminuiu na população adulta, devido às preocupações previamente descritas e ao medo de frequentar estabelecimentos de saúde.^( 17 )^As vacinas pneumocócicas e contra influenza estão sendo também impactadas pela COVID-19. Tem sido observado aumento na incidência e na mortalidade por COVID-19 em pacientes com doenças pulmonares e cardiovasculares crônicas, além de diabetes, sendo todos esses fatores de risco reconhecidos para infecções pneumocócicas.^( 18 )^No entanto, existem poucos dados disponíveis sobre a relação entre influenza e COVID-19.^( 6 , 19 , 20 )^Nos Estados Unidos, a cobertura de vacinas financiadas pelo governo, incluindo as vacinas contra hepatite, meningite, poliomielite e rotavírus, diminuiu drasticamente, em comparação a 2019. Na cidade de Nova Iorque, a diminuição na média geral de vacinação foi de 63%, chegando a 91% para crianças acima dos 2 anos de idade, enquanto no estado da Califórnia a redução foi de 40%.^( 21 )^No Estado de Ohio, a redução na vacinação da população pediátrica foi de 45%. Enquanto, em circunstâncias normais, cerca de mil vacinas contra o sarampo seriam aplicadas mensalmente, em abril de 2020, somente 32 foram aplicadas naquele Estado.^( 22 )^

A hesitação e a recusa vacinal vêm sendo um grave problema de saúde pública já há muitos anos, tanto em período prévio quanto durante a sindemia da COVID-19. Muitas barreiras à vacinação, sendo a maioria centrada em preocupações sobre a segurança das vacinas, são bem descritas na literatura.^( 23 )^Todavia, os motivos para o atraso ou a recusa vacinal mudam com o tempo, conforme evidenciado durante essa sindemia. O alarmismo deve ser combatido com informações legítimas, já que o compartilhamento de notícias e opiniões falsas e sem fundamento em comunidades vulneráveis contribui significativamente para a desconfiança que permeia o desenvolvimento e a adesão às vacinas.^( 24 )^A vacinação está entre as medidas de saúde pública mais eficazes para prevenir a propagação de doenças. Um grande número de ações é requerido para promover a conscientização e combater a desinformação, a fim de que existam a manutenção da cobertura vacinal e a atenuação do risco de infecções preveníveis. Há uma necessidade urgente de incentivo a ações governamentais, apoio às comunidades e tranquilização da população em relação à importância e à segurança da vacinação de rotina em Unidades Básicas de Saúde durante a sindemia da COVID-19. Uma vacina eficaz é provavelmente o método mais importante para controlar e conter a sindemia, e essa opinião é compartilhada por autoridades de saúde pública do mundo todo. No geral, uma vacina efetiva é desenvolvida em, no mínimo, 1 a 2 anos,^( 25 – 28 )^e a existência de uma vacina eficaz é indispensável para a prevenção de morbidade e mortalidade associadas.^( 29 )^Até o momento, dezenas de vacinas candidatas contra o SARS-CoV-2 já foram ou estão sendo testadas em ensaios clínicos,^( 30 )^sendo que algumas já foram aprovadas e estão sendo implementadas em programas de vacinação em massa.^( 31 , 32 )^Entretanto, a Rússia e a China foram acusadas de aplicar antecipadamente vacinas contra o SARS-CoV-2 fora de ensaios clínicos, ação que foi amplamente criticada devido ao perfil de segurança incerto dessas vacinas candidatas, considerando a ausência de dados robustos de segurança e eficácia provindos de ensaios clínicos de fase 3.^( 33 , 34 )^

De acordo com a OMS, uma “clara demonstração de eficácia (de base populacional), idealmente com estimativa pontual de ~50%”, é o padrão mínimo para que uma vacina recém-desenvolvida seja aceita para uso em massa contra a COVID-19. A eficácia dessa vacina deve ser verificada em relação aos seguintes parâmetros de avaliação: “infecção, quadro grave e/ou transmissão viral”.^( 35 )^Também são necessários dados de eficácia de longo prazo e de perfis de segurança. Logo, são requeridos ensaios clínicos com duração de tempo adequado e número suficiente de participantes, para avaliar melhor se as vacinas podem agravar a COVID-19 (em outras palavras, potencializar a doença).^( 36 , 37 )^Novas evidências são, portanto, imprescindíveis para validar o uso das vacinas contra o SARS-CoV-2 e evitar qualquer impacto negativo na aceitação pública dessas vacinas, o que poderia dificultar o controle da propagação da doença, bem como prejudicar a vacinação de rotina contra outras doenças transmissíveis.^( 38 )^

Em conclusão, as autoridades públicas estão estabelecendo processos rápidos de aprovação para vacinas efetivas contra o SARS-CoV-2, e grandes campanhas de vacinação já estão em andamento em vários países. A hesitação vacinal continua sendo um problema persistente, tanto para a vacinação contra o SARS-CoV-2 como para todos os outros programas de vacinação de rotina. Acredita-se que medidas eficazes de saúde pública possam reduzir o impacto do atraso ou a recusa vacinal nos programas de vacinação de rotina, evitando-se o ressurgimento de doenças já erradicadas.
